# Evaluation of ChatGPT as a supplementary tool for pituitary adenomas: An observational study based on simulated consultations

**DOI:** 10.1097/MD.0000000000045928

**Published:** 2025-11-14

**Authors:** Yuhui Chen, Yuyang Chen, Li Chen, Tianshun Feng, Shousen Wang

**Affiliations:** a Department of Neurosurgery, Fuzong Clinical Medical College of Fujian Medical University, Fuzhou, Fujian, China.

**Keywords:** ChatGPT, large language model, patient education, pituitary adenoma, supplementary tool

## Abstract

Chat Generative Pretrained Transformer (ChatGPT), a large language model developed by OpenAI, has shown potential in healthcare communication and patient education. However, its performance in specialized medical domains, such as pituitary adenomas (PAs), remains unclear. Therefore, this study aimed to evaluate the reliability and consistency of ChatGPT in answering PA-related questions. We hypothesized that ChatGPT would demonstrate high reliability in responding to general patient-oriented queries but lower reliability for specialized clinical questions. A total of 256 PA-related questions were collected from patients and families, clinical practice guidelines, and medical question banks. Each question was input into ChatGPT (GPT-4, March 2025 version), and the generated responses were independently reviewed by 2 senior neurosurgeons. Any discrepancies in their assessments were resolved by a third neurosurgeon with over 30 years of clinical experience. Responses were categorized as completely correct, partially correct but usable, partially correct, or incorrect. Responses rated as completely correct or partially correct but usable were considered reliable. Consistency was assessed based on the stability of response quality across similar question types. Comparisons were made by question type (general vs professional) and source using univariate analysis. Among the 256 responses, 143 (55.8%) were completely correct, 68 (26.6%) were partially correct but usable, 19 (7.4%) were partially correct, and 26 (10.2%) were incorrect. Overall, 82.4% of the responses were considered reliable, and 68.4% demonstrated consistency. Reliability was significantly higher for general questions than for professional ones (95.0% vs 78.6%, OR = 5.182, 95% CI: 1.545–17.378, *P* = .003), and for guideline-derived questions compared to question bank-derived ones (100.0% vs 75.7%, OR = 1.321, 95% CI: 1.214–1.437, *P* = .017). Differences in consistency across subgroups were not statistically significant. ChatGPT exhibits high reliability and moderate consistency in answering PA-related questions, especially for general and guideline-based content. It may serve as a supplementary source of patient information but should not replace professional medical consultation, particularly in complex or surgical contexts. As this study was conducted in an artificial testing environment without validation in real patient consultations, the generalizability of the findings remains limited.

## 1. Introduction

In recent years, machine learning and artificial intelligence have advanced rapidly. On November 30, 2022, OpenAI released a chatbot called Chat Generative Pretrained Transformer (ChatGPT), which has attracted global attention. ChatGPT is a large language model based on the GPT-4 architecture. This model has a large parameter size and powerful language generation capabilities. Through deep pretraining and self-supervised learning, it learns language patterns and structures from large-scale unsupervised data, enabling it to understand and generate human-like natural languages.^[1]^

Data in ChatGPT are limited to 2023; however, it demonstrates great potential in the medical field.^[2]^ For example, it can provide evidence-based answers to public health questions,^[3]^ assist in various medical exams,^[4–6]^ and serve as a support tool for breast cancer decision-making.^[7]^ ChatGPT has provided impressive results in neurosurgery multiple-choice medical board examinations^[8]^ and predominantly in higher-order management questions;^[9]^ however, to our knowledge, no study has evaluated the reliability and consistency of ChatGPT in response to neurosurgery-specific disease questions.

Pituitary adenoma (PA) is the most common tumor of the sellar region and affects approximately 10% of the general population. It often causes endocrine disorders and visual field defects, severely impacting the quality of life.^[10]^ Due to the heterogeneity of these tumors, different types of PAs can present with various clinical symptoms.^[11]^ The internet can provide disease-related knowledge; however, the complexity of the literature, possible errors in information, and difficulties in describing the condition using professional terminology can confuse and obscure patients’ understanding of the disease. ChatGPT can address these limitations by providing a convenient platform for patients to obtain relevant information through simple conversations.^[12]^ Therefore, we believe patients with PAs can utilize ChatGPT as an information source. Nevertheless, concerns have been raised that ChatGPT may generate inaccurate or fabricated information (“hallucinations”), provide responses without verifiable source citations, and demonstrate variability in response quality across repeated queries. Furthermore, ChatGPT has not received extensive and specialized medical training data, and prior studies have shown that its responses may include false or misleading references.^[13–15]^ Importantly, recent work has emphasized that such risks may be amplified in rare or less common medical conditions, where limited high-quality training data make hallucinations more likely.^[16–19]^ Ilić and Sarajlija ^[16]^ reported that artificial intelligence in the diagnosis of pediatric rare diseases is particularly prone to misinformation due to data scarcity and atypical clinical presentations. These limitations underscore the importance of critically evaluating ChatGPT before considering its integration into patient education or clinical workflows.

Therefore, determining the reliability of this new tool is crucial. We hypothesized that ChatGPT would demonstrate high reliability in answering general patient-oriented queries but significantly lower reliability when addressing specialized clinical questions related to PAs.

## 2. Materials and methods

We collected and analyzed responses from GPT-4 (OpenAI, San Francisco). All the questions were collected on March 20, 2025. This study did not involve human participants or identifiable data; therefore, ethical committee approval was not required.

**Figure 1.**
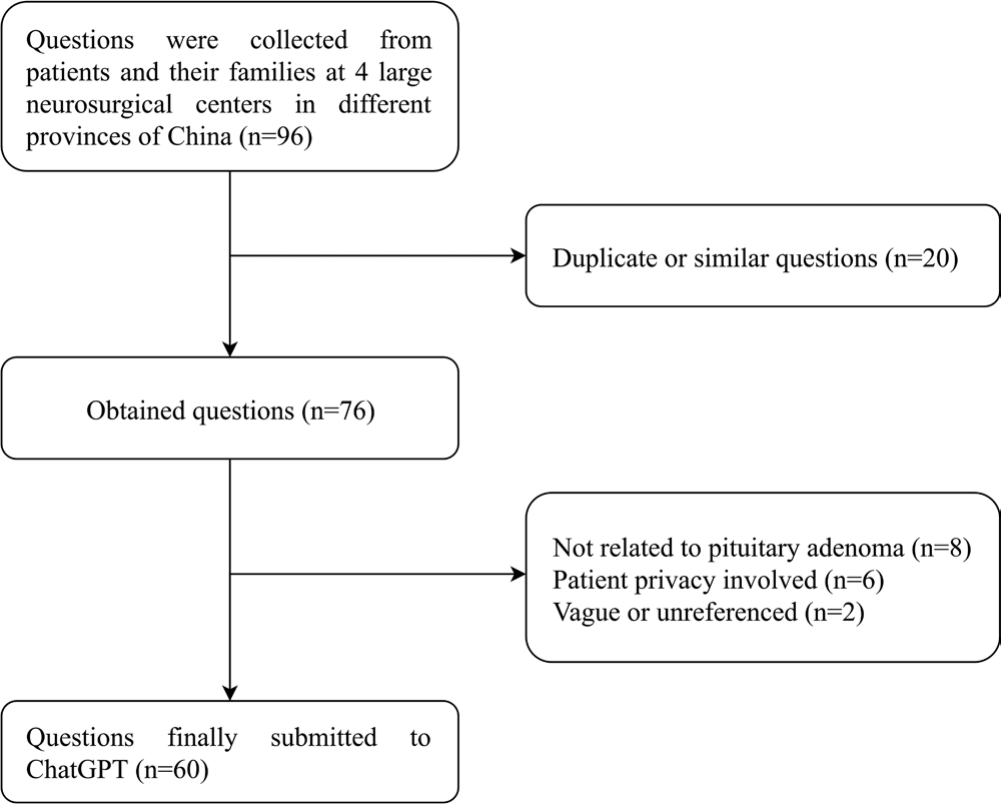
Flow chart of collecting questions from patients and their families.

To increase the representativeness of questions related to patients with pituitary adenoma and their families, we collected relevant questions from 4 large neurosurgical centers in different provinces of China. After excluding duplicate or similar questions, we modified the grammar to ensure accuracy while preserving the intended meaning. All questions were then entered into ChatGPT in English, with patient-derived questions translated from Chinese into English to ensure clarity and correctness. Finally, 60 questions related to PAs were collected (Fig. 1).

To evaluate the performance of ChatGPT in terms of knowledge and management measures related to pituitary incidentaloma and aggressive pituitary tumors, we formulated 6 questions about pituitary incidentaloma and 17 questions about aggressive pituitary tumors based on the guidelines.^[20,21]^ We used the guideline recommendations as standard answers.

To further examine the knowledge base of ChatGPT and its ability to address clinical issues, we included 153 open-ended and 20 multiple-choice questions based on published question banks.^[22–24]^

We categorized questions from patients and their families as general questions (n = 60) and questions from guidelines and question banks as professional questions (n = 196). Based on the question topic, we divided them into questions on basic knowledge of PAs (n = 71) and clinically related questions, including diagnosis and assessment (n = 50), radiological examination (n = 44), pharmacological treatment (n = 15), surgical management (n = 31), and postoperative management and follow-up (n = 45).

Given that the questions posed in this study relied on many ordinary patients, we accessed ChatGPT (March 25 version; OpenAI). This version of ChatGPT is based on GPT-4, one of the largest language models. It is pretrained on vast textual data, enabling it to generate responses to various text-based inputs.

**Figure 2.**
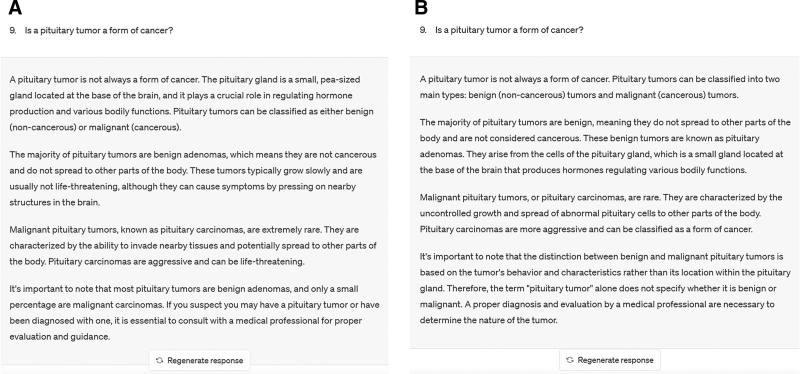
The response of ChatGPT to the same question twice. (A) In the first response, Reviewer 1 and Reviewer 2 rated it as correct; Reviewer 3 did not evaluate it, resulting in a correct final assessment. (B) In the second response, Reviewer 1 and Reviewer 2 rated it as correct; Reviewer 3 did not evaluate it, resulting in a correct final assessment. Therefore, the response of ChatGPT to the question is reliable and consistent. ChatGPT = Chat Generative Pretrained Transformer.

Each question was inputted using “New Chat.” For open-ended questions, the question stem was inputted into ChatGPT, whereas for multiple-choice questions, the question stem and its multiple options were inputted into ChatGPT. Multiple-choice answers were evaluated against guideline recommendations and expert consensus. Each question was inputted into ChatGPT twice, and 2 generated responses were recorded. These responses were independently reviewed by 2 senior neurosurgeons with advanced professional titles. They assessed the accuracy of each response and categorized it as correct, partially correct but usable, a combination of correct and incorrect, or completely incorrect. Any discrepancies between the 2 reviewers in their categorizations were reviewed and resolved by a third neurosurgeon with over 30 years of clinical experience in neurosurgery. Responses categorized as correct or partially correct but usable were classified as reliable, whereas responses categorized as a combination of correct and incorrect, or completely incorrect were classified as unreliable. In this study, consistency was defined as the agreement between the final assessments of 2 or 3 reviewers of the 2 responses from ChatGPT, regardless of whether the response was correct or incorrect. Examples of ChatGPT’s repeated responses and reviewer assessments are illustrated in Figure 2, demonstrating how reliability and consistency were evaluated across different outputs.

This study compared performance in terms of reliability and consistency between the source of the questions (general vs professional) and the topic of the questions (basic vs clinically related). In addition, a subgroup analysis was conducted with clinically relevant questions. Odds ratios (ORs) with 95% confidence intervals (CIs) were calculated to quantify effect sizes. All statistical analyses were performed using SPSS version 27.0, with statistical significance set at *P* < .05.

## 3. Results

We inputted 256 questions related to PAs. ChatGPT provided correct responses for 143 questions (55.8%), partially correct but usable responses for 68 questions (26.6%), a combination of correct and incorrect responses for 19 questions (7.4%), and completely incorrect responses for 26 questions (10.2%). Regarding reliability, 211 responses (82.4%) were categorized as reliable, whereas 45 (17.6%) were categorized as unreliable. Consistency was observed among 175 responses (68.4%).

**Figure 3.**
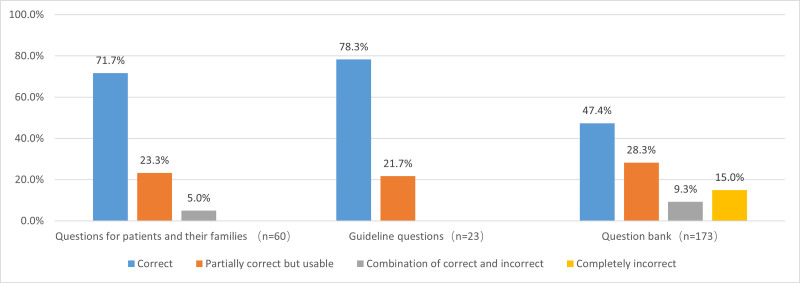
Accuracy assessment grading percentages of the responses of ChatGPT to pituitary adenoma questions from different sources. ChatGPT = Chat Generative Pretrained Transformer.

Figure 3 illustrates the accuracy of ChatGPT responses by question source. ChatGPT performed worst on question bank items, with fewer than half answered correctly (47.4%) and 15.0% completely incorrect. In contrast, higher accuracy was observed for patient-based and guideline-based questions, with 71.7% and 78.3% correct responses, respectively, and no completely incorrect answers. In terms of reliability, general questions were significantly more reliable than professional questions (95.0% vs 78.6%, OR = 5.182, 95% CI: 1.545–17.378, *P* = .003). Similarly, guideline-based questions were more reliable than bank-based questions (100.0% vs 75.7%, OR = 1.321, 95% CI: 1.214–1.437, *P* = .017) (Table 1). No significant differences were observed in consistency between general and professional questions (73.3% vs 66.8%, OR = 1.365, 95% CI: 0.716–2.600, *P* = .344) or between guideline- and bank-based questions (82.6% vs 64.7%, OR = 2.587, 95% CI: 0.842–7.948, *P* = .087) (Table 2).

**Table 1:** Reliability of ChatGPT responses to questions from different sources and on different topics.

Question types	Reliable, n (%)	Unreliable, n (%)	OR	95% CI	*P*
All questions (n = 256)	154 (82.4)	45 (17.6)			
Classified by source			5.182	1.545–17.378	.003
General questions	57 (95.0)	3 (5.0)			
Patients and their families (n = 60)	57 (95.0)	3 (5.0)			
Professional questions	154(78.6)	42 (21.4)	1.321	1.214–1.437	.017
Guideline (n = 23)	23 (100.0)	0 (0.0)			
Question bank (n = 173)	131 (75.7)	42 (24.3)			
Classified by topic			0.639	0.323–1.266	.197
Basic questions	55 (77.5)	16 (22.5)			
Basic knowledge (n = 71)	55 (77.5)	16 (22.5)			
Clinically relevant questions	156 (84.3)	29 (15.7)	–	–	.504
Diagnosis and assessment (n = 50)	43 (86.0)	7 (14.0)			
Radiological examination (n = 44)	35 (79.5)	9 (20.5)			
Pharmacological treatment (n = 15)	14 (93.3)	1 (6.7)			
Surgical management (n = 31)	24 (77.4)	7 (22.6)			
Postoperative management and follow-up (n = 45)	40 (88.9)	5 (11.1)			

ChatGPT = Chat Generative Pretrained Transformer.

**Table 2:** Consistency of ChatGPT responses to questions from different sources and on different topics.

Question types	Consistency, n (%)	Inconsistency, n (%)	OR	95% CI	*P*
All questions (n = 256)	175 (68.4)	81 (31.6)			
Classified by source			1.365	0.716–2.600	.344
General questions	44 (73.3)	16 (26.7)			
Patients and their families (n = 60)	44 (73.3)	16 (26.7)			
Professional questions	131 (66.8)	65 (33.2)	2.587	0.842–7.948	.087
Guideline (n = 23)	19 (82.6)	4 (17.4)			
Question bank (n = 173)	112 (64.7)	61 (35.3)			
Classified by topic			1.680	0.901–3.134	.101
Basic questions	54 (76.1)	17 (23.9)			
Basic knowledge (n = 71)	54 (76.1)	17 (23.9)			
Clinically relevant questions	121 (65.4)	64 (34.6)	-	-	.662
Diagnosis and assessment (n = 50)	36 (72.0)	14 (28.0)			
Radiological examination (n = 44)	26 (51.9)	18 (48.1)			
Pharmacological treatment (n = 15)	11 (73.3)	4 (26.7)			
Surgical management (n = 31)	19 (61.3)	12 (38.7)			
Postoperative management and follow-up (n = 45)	29 (64.4)	16 (35.6)			

ChatGPT = Chat Generative Pretrained Transformer.

**Figure 4.**
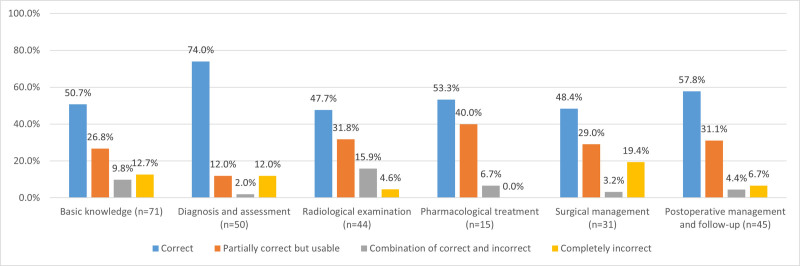
Accuracy assessment grading percentages of the responses of ChatGPT to pituitary adenoma questions from different topics. ChatGPT = Chat Generative Pretrained Transformer.

Figure 4 illustrates the accuracy of ChatGPT responses by question topics. The “diagnosis and assessment” had the highest correct response rate (74.0%), whereas “surgical management” showed the most completely incorrect responses (19.4%). Notably, “pharmacological treatment” had no completely incorrect answers. When grouped by clinical topic, reliability ranged from 77.4% (surgical management) to 93.3% (pharmacological treatment). However, there was no significant difference in reliability between basic and clinically relevant questions (77.5% vs 84.3%, OR = 0.639, 95% CI: 0.323–1.266, *P* = .197), nor among the subgroups of clinically relevant questions (*P* = .504) (Table 1). No significant difference was observed in consistency between the basic (OR = 1.680, 95% CI: 0.901–3.134, *P* = .101) and clinically relevant questions or among the subgroups of clinically relevant questions (*P* = .662) (Table 2).

## 4. Discussion

In this prospective exploratory study, we evaluated ChatGPT’s ability to address questions related to PAs. Our findings indicate that ChatGPT provided generally accurate and comprehensible responses, with a reliability rate exceeding 80%, while the overall consistency was moderate at approximately 70%. Nonetheless, its performance was less robust for more specialized clinical questions. These limitations highlight the need for further refinement and training to improve both reliability and consistency before ChatGPT can be considered for broader application in patient education or clinical contexts.

A recent study^[4]^ examined the performance of ChatGPT in the United States Medical Licensing Examination. ChatGPT achieved scores ranging from 52.4 to 75.0% across the 3 examinations, thus reaching or approaching the passing threshold. This indicates the potential for medical education and clinical decision support. Hopkins et al^[8]^ evaluated the performance of ChatGPT in answering Self-Assessment Neurosurgery questions. ChatGPT performed well, although its performance was lower than that of regular Self-Assessment Neurosurgery users, better than that of medical students, and slightly lower than that of resident physicians currently preparing for the exam. In our study, ChatGPT provided reliable responses to 82.4% of the pituitary adenoma questions, demonstrating its good performance in the subspecialty of PAs, which aligns with its performance in the United States Medical Licensing Examination and Self-Assessment Neurosurgery. However, the reliability of its responses to general questions was higher than that of professional questions, indicating its potential for responding to patients’ daily questions but limitations in specialized aspects. Its performance in guideline questions was better than that in question bank questions, possibly owing to the generic nature of guideline question answers, whereas question bank questions require an analysis of the questions and consideration of various other data sources. Therefore, ChatGPT performed poorly in question bank questions but provided 75.7% reliable responses. Further exploration and testing of its performance in a pituitary adenoma question bank is needed in the future.

In terms of consistency, the performance of ChatGPT was moderate, with an overall consistency of 68.4%, which was lower than its performance in areas such as liver cirrhosis, hepatocellular carcinoma,^[12]^ bariatric surgery,^[25]^ and lung cancer.^[26]^ Yeo et al^[12]^ evaluated the performance of ChatGPT in response to questions related to liver cirrhosis and hepatocellular carcinoma. They categorized the responses into 2 groups (1 and 2 vs 3 and 4); if the rating of each response belonged to the same group, the 2 responses for each question were considered consistent. The consistencies for liver cirrhosis and hepatocellular carcinoma responses were 90.4% and 98.6%, respectively. Rahsepar et al^[26]^ evaluated the performance of ChatGPT and Google Bard in responding to common lung cancer questions; ChatGPT had a consistency of 90%, while that of Google Bard was 57.5%. Their approach merged ratings into broader categories, which may have increased the reported consistency by tolerating partial agreement.^[12,26]^ In contrast, our study required reviewers to assign both outputs to exactly the same category across 4 more detailed classifications, representing a stricter and more conservative definition of consistency. These differences underscore the critical impact of methodological factors, including scoring criteria and the definition of consistency, on reported outcomes. Therefore, there is an urgent need for a standardized framework to evaluate consistency, ensuring valid and reproducible cross-study comparisons.

In this study, ChatGPT showed reasonable performance in addressing pituitary adenoma-related questions, with potential as a supplementary source of information. However, its accuracy and consistency remain limited, and its outputs cannot substitute professional medical consultation. The responses sometimes varied and included incorrect or false information, with 17.6% of answers in our study classified as unreliable. Such errors pose potential dangers if patients rely on ChatGPT without direct medical supervision, as seemingly credible but inaccurate responses may mislead decision-making. With the advancement of technology that makes medical knowledge more accessible, patients increasingly turn to search engines and ChatGPT for health information. Nevertheless, critical risks remain: ChatGPT cannot provide personalized recommendations based on regional guideline differences, nor can it ensure accuracy across diverse clinical contexts. These limitations underscore the need for cautious interpretation of its outputs and confirm that ChatGPT should not be regarded as a consultation tool at this stage. Instead, it may serve only as a supplementary source of health information under professional oversight. Future directions should include improving its training data, ensuring alignment with updated guidelines, and validating patient comprehension and trust through real-world usability studies. In addition, future research should broaden its scope to include multi-model comparisons (e.g., ChatGPT, Google Bard, DeepSeek), evaluation of multilingual performance, and integration with verified medical databases to enhance accuracy, reliability, and applicability in diverse clinical settings.

This study had certain limitations. To our knowledge, no dedicated question bank or examination has been established for PAs. Therefore, we selectively inputted pituitary adenoma-related questions from various question banks into ChatGPT. Additionally, although the questions from patients and their family members were collected from large neurosurgical centers in 4 different provinces, potential bias remains in selecting these questions. This sampling approach, while ensuring clinical relevance, may not fully represent the diversity of patient concerns in other regions or healthcare settings. In the future, more systematic and geographically diverse methods for question selection should be considered to enhance representativeness. Second, no standardized criterion exists for grading the accuracy of ChatGPT responses, leading to significant variations among studies. Therefore, a standardized and objective scoring system must be proposed to ensure the reliability of the responses and reduce heterogeneity among different studies. Furthermore, we adopted a stricter definition of consistency, requiring identical reviewer categorization of both outputs. While this provides a rigorous and conservative measure of model stability, it may limit comparability with prior studies that used broader definitions. Third, this study was conducted in an artificial testing environment based on simulated consultations, and the findings have not yet been validated in real patient interactions. Future studies incorporating patient validation are warranted to confirm the applicability of these results in clinical practice. Finally, considering that ChatGPT is the most popular free artificial intelligence chatbot used by most of the population, we evaluated the reliability and consistency of ChatGPT in responding to pituitary adenoma-related questions to understand the strengths and limitations of the public version. However, DeepSeek and Google Bard is gaining popularity among the public. Future research may involve comparing the differences between these models.

In conclusion, ChatGPT demonstrates potential as a supplementary source of medical information for patients. However, its responses occasionally contain inaccuracies or misleading content, highlighting the risk of misinformation. Therefore, ChatGPT should not be regarded as a substitute for professional medical consultation, particularly in complex or surgical contexts. Careful oversight by qualified healthcare professionals remains essential before integrating such tools into clinical practice.

## Acknowledgments

The authors thank all institutions and individuals who supported this research.

## Author contributions

**Conceptualization:** Yuhui Chen, Shousen Wang.

**Data curation:** Li Chen, Tianshun Feng.

**Formal analysis:** Yuyang Chen, Li Chen.

**Investigation:** Yuhui Chen, Yuyang Chen, Li Chen, Tianshun Feng.

**Methodology:** Yuhui Chen, Yuyang Chen.

**Supervision:** Shousen Wang.

**Visualization:** Li Chen, Tianshun Feng.

**Writing – original draft:** Yuhui Chen.

**Writing – review & editing:** Shousen Wang.
